# Toward non-enzymatic glucose sensing based on organic electronics: organic field-effect transistors with multifunctionalized extended-gate electrodes

**DOI:** 10.1007/s44211-026-00892-3

**Published:** 2026-03-30

**Authors:** Yui Sasaki, Muthukumar Govindaraj, Tsuyoshi Minami

**Affiliations:** 1 Research Center for Advanced Science and Technology, The University of Tokyo, 4-6-1 Komaba, Meguro-ku, Tokyo, Japan; 2 Institute of Industrial Science, The University of Tokyo, 4-6-1 Komaba, Meguro-ku, Tokyo, Japan; 3 JST, PRESTO, 4-1-8 Honcho, Kawaguchi, Saitama Japan

**Keywords:** Organic field-effect transistor, Glucose, Phenylboronic acid, Real-sample analysis, Microfluidics

## Abstract

Accurate glucose analysis has been a major research focus for the past five decades since the pioneering development of enzymatic glucose sensing, driven by the global demand for rapid and reliable diagnostics. In contrast to well-established enzymatic glucose sensors, the development of chemical sensors based on supramolecular receptors remains a developing area. Among the supramolecular receptors for glucose, phenylboronic acid (PBA) derivatives provide unique functions, such as the formation of boronate esters with glucose and their reversibility. In this study, we focus on organic field-effect transistors (OFETs) combined with PBA derivatives as chemical sensors. Although the beneficial properties of OFETs have been explored for physical sensor applications in organic electronics, the instability of organic devices remains a major limitation for chemical sensing applications. To address this issue, an extended-gate structure was employed as the OFET-based chemical sensor configuration. In this sensor design, an extended-gate electrode functionalized with a PBA derivative self-assembled monolayer was connected to the gate electrode of the OFET, enabling glucose detection via boronate ester formation at the aqueous-sensing electrode interface. In this mini-review, we introduce strategies for establishing non-enzymatic glucose sensors using OFETs with multifunctionalized extended-gate electrodes. Notably, selective glucose detection in human blood plasma has been demonstrated by combining a two-dimensional material that suppresses the physical adsorption of endogenous proteins onto an extended-gate electrode. Finally, an approach that integrates a microfluidic chamber into an extended-gate-based OFET device is introduced to highlight the inherent reversibility of the PBA derivative.

**Graphical abstract:**

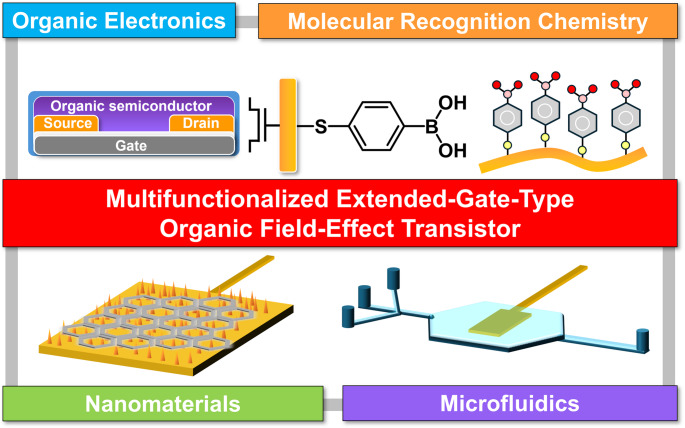

## Introduction

Saccharides are abundant biomolecules that play vital roles in biological systems [1]. Among them, glucose serves as a primary energy source for cellular metabolism in living organisms [2]. In addition, the regulation of blood glucose within a physical range is essential for metabolic homeostasis. Thus, the development of easy-to-use glucose sensors is in high demand in clinical applications. In particular, methods for glucose analysis in human biofluids, such as saliva, sweat, urine, and tears, have been widely explored for non-invasive health monitoring [3]. Traditional glucose detection relies on enzyme-based electrochemical sensors because of the lock-and-key principle for highly selective detection [4, 5]. However, the limited range of detectable analytes provided by enzyme libraries has accelerated the development of non-enzymatic glucose sensors that enable design based on molecular recognition chemistry [6–10].

**Fig. 1:**
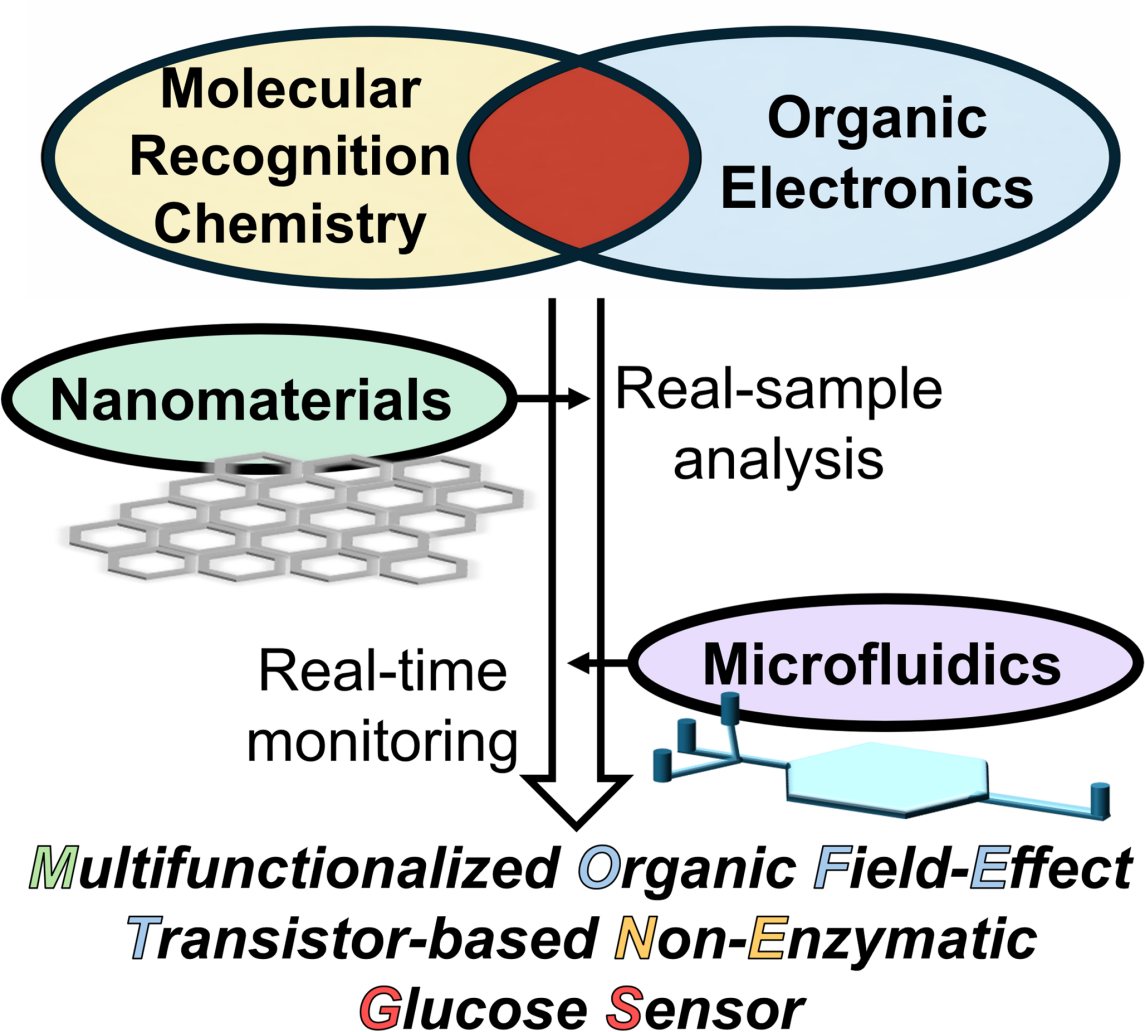
Illustrated concept of non-enzymatic glucose sensors based on organic field-effect transistors (OFETs) with multifunctionalized extended-gate electrodes

In the field of molecular recognition chemistry, saccharide receptors have been vigorously developed and applied to selective glucose sensing for diagnosis [7, 8]. Among them, phenylboronic acid (PBA) is a representative saccharide receptor that forms a boronate ester with a *cis*-diol moiety through a dynamic covalent bond [10, 11]. One of the unique properties of PBA derivatives is the cross-reactive response to derivatives with *cis*-diol moieties [9], enabling simultaneous detection based on pattern recognition [11, 12]. However, the inherent cross-reactivity of PBA poses a challenge for achieving highly selective glucose detection over structurally similar saccharides. Focusing on the selective detection mechanisms in Mother Nature, enzymes and antibodies recognize specific analytes through multiple non-covalent interactions [13]. In other words, supramolecular interactions contribute to providing specificity [13]. Indeed, supramolecular receptors incorporating multiple PBA moieties have achieved selective glucose sensing [14]. In this system, the multivalent interactions between the receptor and glucose played key roles in the discrimination of the analyte from the analogues. Based on this, we have focused on self-assembled monolayers (SAMs). In the concept of SAMs, molecules spontaneously align on solid substrates to form nanoscale monolayer surfaces with various functions [15, 16]. Notably, SAMs functionalized with terminal recognition moieties allow chemical sensing at interfaces between aqueous media and the solid substrates [17–19]. The large surface area of the SAM-attached electrode participates in unique analyte recognition derived from multivalent interactions among SAMs, whereby the selective analyte recognition can be demonstrated [17–19]. In addition, the reversible recognition fashion of the PBA derivatives is also an inherent feature of dynamic covalent bonds [20]. Along with these attractive features of the PBA derivatives, various sensing applications have been reported in the supramolecular chemistry field, while glucose sensing at the solid–liquid interface remains challenging, due to difficulties in device implementation. To this end, we focused on integrating organic electronics technologies into supramolecular recognition mechanisms [21]. Among these, organic field-effect transistors (OFETs) have emerged as promising platforms for chemical sensing, owing to their advantages, such as solution-processability and compatibility with flexible substrates [22, 23]. According to the detection principle in an OFET-based chemical sensor, a gate electrode is used as the sensing portion, as described below. Therefore, a PBA derivative (i.e., 4-mercaptophenylboronic acid) can be immobilized as SAMs on the sensing electrode, which allows selective glucose detection at the interface between the gate electrode and aqueous media [17–19]. However, the instability of organic layers in OFETs is a concern for chemical sensing applications that require operation in aqueous media [24]. In contrast, an extended-gate-type OFET configuration that isolates the transistor unit from the sensing electrode enables stable device operation upon analyte capture [21, 22, 25, 26]. Moreover, the extended-gate surface can be functionalized depending on the sensing purpose, which broadens the potential of the OFETs as chemical sensors for practical applications, such as real-sample analysis and real-time monitoring. In general, the interference effects from endogenous proteins suppress sensor performance; thus, surface engineering of the sensing electrode is necessary. To address this, we propose an approach using a two-dimensional material on the extended-gate electrode surface to suppress the physical adsorption of the interferent proteins in human body fluids onto the sensing electrode [18]. Furthermore, an integrated design of a microchamber into the extended-gate electrode is introduced to demonstrate the applicability of continuous glucose detection by the reversible behavior of the PBA derivative [19]. Overall, this mini-review provides an overview of non-enzymatic glucose detection using OFETs and multifunctionalized extended-gate electrodes. In particular, we focused on approaches to endow multiple functions onto an extended-gate surface based on fused technology, including molecular recognition chemistry, nanomaterials, and microfluidics (Fig. 1).

## Design and principle of the extended gate-type OFET-based chemical sensor

**Fig. 2:**
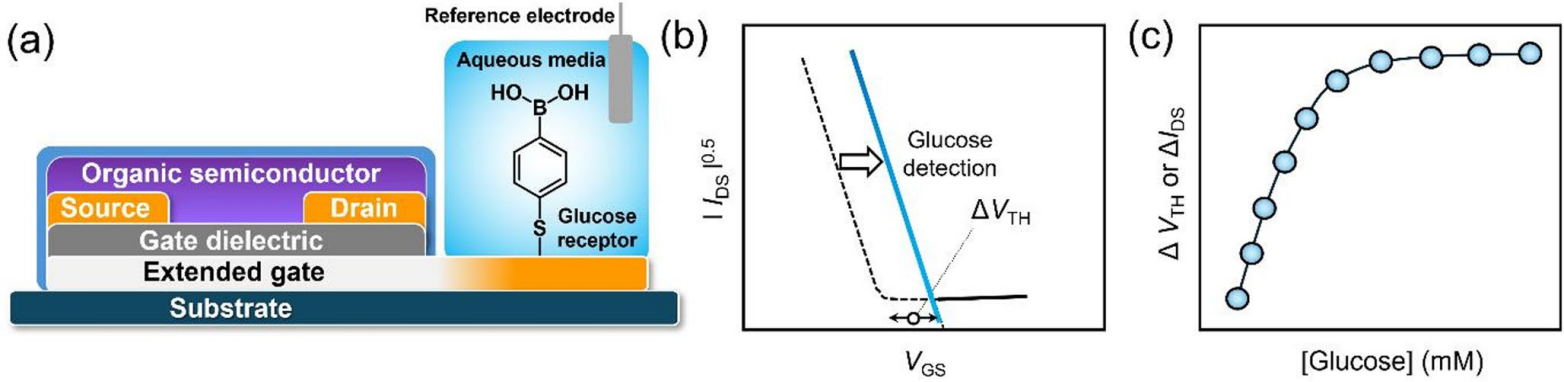
**a** Illustrated configuration of an extended-gate-type organic field-effect transistor (OFET) functionalized with 4-mercaptophenylboronic acid for glucose sensing. **b** Transistor characteristics upon analyte detection. The term *V*_TH_ indicates the threshold voltage. **c** Correlation between glucose concentrations and changes in transistor characteristics (Δ*V*_TH_ or Δ*I*_DS_). The terms Δ*V*_TH_ and Δ*I*_DS_ represent changes in threshold voltage and drain current, respectively

FETs are three-terminal devices comprising a source, drain, and gate electrode, where an electric field applied through the gate dielectric modulates the charge transport within a semiconductor layer [27]. Since their invention in the 1980s, [28] OFETs have been extensively applied to flexible displays and sensor devices [21, 22, 29, 30]. In contrast to well-established physical sensors, the development of OFET-based chemical sensors is still under development. Among the OFET configurations for chemical sensing applications, the extended-gate structure is the focus of this mini-review. The device structure allows analyte capture on the surface of an external sensing electrode (referred to as the extended-gate electrode) that is connected to the gate electrode of an OFET device (Fig. 2a). In general, the extended-gate structure is classified into two types: integrated and separated [22]. The integrated structure enables the fabrication of both the OFET unit and the sensing electrode on a single chip, thereby broadening the application of OFET devices [31]. Meanwhile, the separated extended-gate electrode is used as a disposable sensing unit in real-sample analysis, taking hygiene considerations into account. The surface of the extended-gate electrode was functionalized with 4-mercaptophenylboronic acid as the SAM, through Au–S bonds. With this electrode design, the target glucose can be detected at the interface between the extended-gate electrode and aqueous media containing the analyte [17]. The superior tunability of monolayer formation based on molecular designs enables precise surface engineering, enhancing the accuracy of interfacial chemical sensing. In this regard, the surface of the extended-gate electrode functionalized with the PBA derivative is comprehensively characterized using instrumental assessment methods [17–19]. For example, photoelectron yield spectroscopy in air is used to estimate the work function of the extended-gate electrode functionalized with SAMs, which corresponds to the electron-withdrawing or donating properties of the immobilized SAMs [32, 33]. Infrared spectroscopy allows for the estimation of not only the functional groups but also the orientation of SAMs [34, 35]. In addition, X-ray photoelectron spectroscopy is performed to obtain elemental information derived from the components of SAMs [36]. The wettability profiles of the electrode surface indicate the hydrophilicity of SAMs [37].

During sensor development, appropriate device configurations, materials, and fabrication processes are necessary for achieving reproducible device performance in practical glucose-sensing applications. Device operation at low voltages is required for sensing in aqueous media for clinical applications. To this end, thin-dielectric layers combined with a metal oxide and an organic monolayer have been applied for OFET fabrication [38]. Moreover, solution-processable organic semiconductors (e.g., poly{2,5-bis(3-alkylthiophen-2-yl)thieno[3,2-*b*]thiophene} (PBTTT) [39], and derivatives of benzothieno[3,2-*b*][1]benzothiophene (BTBT) [40] and dinaphtho[2,3-*b*:2’,3’-*d*]thiophene (DNT–V) [41] have facilitated the fabrication of organic electronic devices using wetting processes with high reproducibility. Considering the sensing situation under ambient conditions, the surface of the organic semiconductor layer was passivated using hydrophobic materials to enhance mechanical robustness and high durability [42]. After optimization of the device fabrication and characterization of the device performance, the combination of the OFET device and the extended-gate electrode was applied to glucose sensing.

Based on the detection principle of the OFET-based chemical sensor, the shift in the surface potential of the electrode is caused by changes in the charges and dipoles of the immobilized receptors [43]. In the case of glucose sensing, PBA forms a cyclic boronate ester with glucose, accompanied by a transformation of boron from a trigonal to a negatively charged tetrahedral configuration [44, 45]. This interfacial binding event induces a shift in the surface potential of the sensing electrode [46], thereby modulating the characteristics of the OFET device. The changes in the surface potential derived from glucose detection further influenced the conductance of the OFETs, resulting in variations in the drain currents (*I*_DS_s) and threshold voltages (*V*_TH_s) (Fig. 2b). As a result, the estimated Δ*I*_DS_ and Δ*V*_TH_ correspond to sensor responses through transistor characteristics qualitatively and quantitatively (Fig. 2c). The details of the device principle have been summarized in previous review articles [21, 22, 26, 27].

## Sensing performance of the extended-gate-type OFET for selective glucose detection

The advantage of supramolecular receptors is their cross-reactive recognition ability against similar structural analytes [47, 48]. Such unique receptor properties contribute to simultaneous detection, as observed in mammalian olfactory systems [49]. Meanwhile, the inherent cross-reactivity of supramolecular receptors is a bottleneck in selective glucose detection. In contrast, approaches based on the supramolecular interactions of SAMs demonstrate selective analyte recognition. This section introduces a SAM-based OFET chemical sensor for selective glucose detection based on multivalent binding events.

**Fig. 3:**
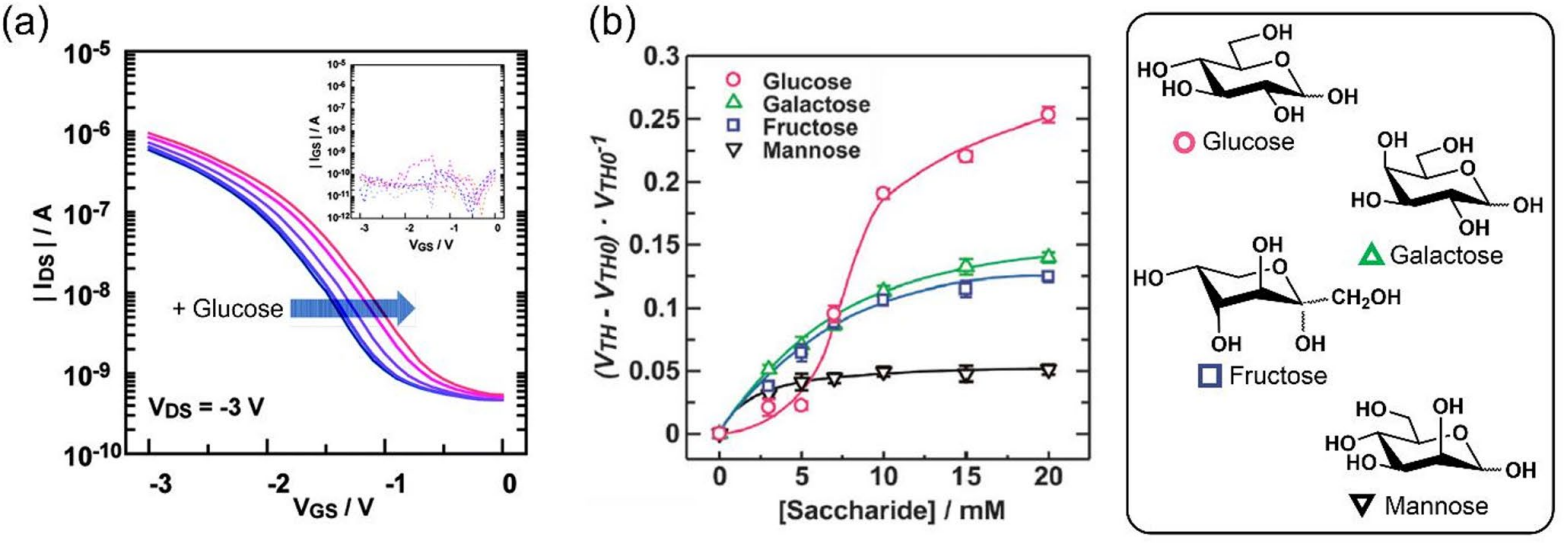
**a** Changes in transfer curves of the extended-gate-type organic field-effect transistor (OFET) functionalized with the phenylboronic acid (PBA) derivative upon glucose addition (0–20 mM). **b** Titration isotherms against glucose (pink circles), galactose (green triangles), fructose (blue squares), and mannose (black inverted triangles). The terms *V*_TH0_ and *V*_TH_ represent the threshold voltages before and after the addition of saccharides. Reproduced with permission from reference [17]. Copyright 2014 The Royal Society of Chemistry

In the OFET fabrication, the π-conjugated polymer material, PBTTT, was used for the semiconductive layer, due to its solution-processability and reproducible device performance [39]. In addition, the double-gate dielectric layer made of a metal oxide layer and a long alkyl chain-linked phosphonic acid was employed to achieve low-voltage operation ( <|3| V) [38]. According to the detection principle of the extended-gate-type OFET, the analyte capture phenomenon in the vicinity of the electrode surface can be sensitively detected [43]. With this strategy, 4-mercaptophenylboronic acid with short molecular length was immobilized on the extended-gate electrode [17]. As shown in Fig. 3a, positive *V*_TH_ shifts were observed with increasing glucose concentration, which can be explained by the formation of negatively charged boronate ester on the electrode surface [17]. From a diagnostic perspective, the OFET-based sensor detected glucose concentrations above 5 mM, corresponding to the physiological range relevant to diabetes monitoring (i.e., normal fasting < 6.1 mM and diabetic ≥ 7.0 mM) [50]. In addition, the device exhibited selective detection of glucose over other saccharides, according to the following orders: glucose > galactose ≈ fructose > mannose (Fig. 3b). Fructose generally exhibits a higher binding affinity toward PBA than glucose in a homogeneous solution owing to a favorable 1:1 binding mode. The distinctly higher response to glucose was attributed to the formation of bis(boronate) complexes between glucose and multiple PBA groups, in contrast to the weaker 1:1 complexes formed with other saccharides. Indeed, reducing the surface density of PBA resulted in the disappearance of the sigmoidal glucose response [17]. In other words, multivalent interactions between the PBA-based SAM and the target saccharides effectively contributed to the discrimination of glucose from other saccharides. Overall, this demonstration revealed the applicability of the combination of the extended-gate-type OFET and the PBA-based SAM for selective glucose sensing.

## Suppression of interfering effects by the combination of a 2D material

**Fig. 4:**
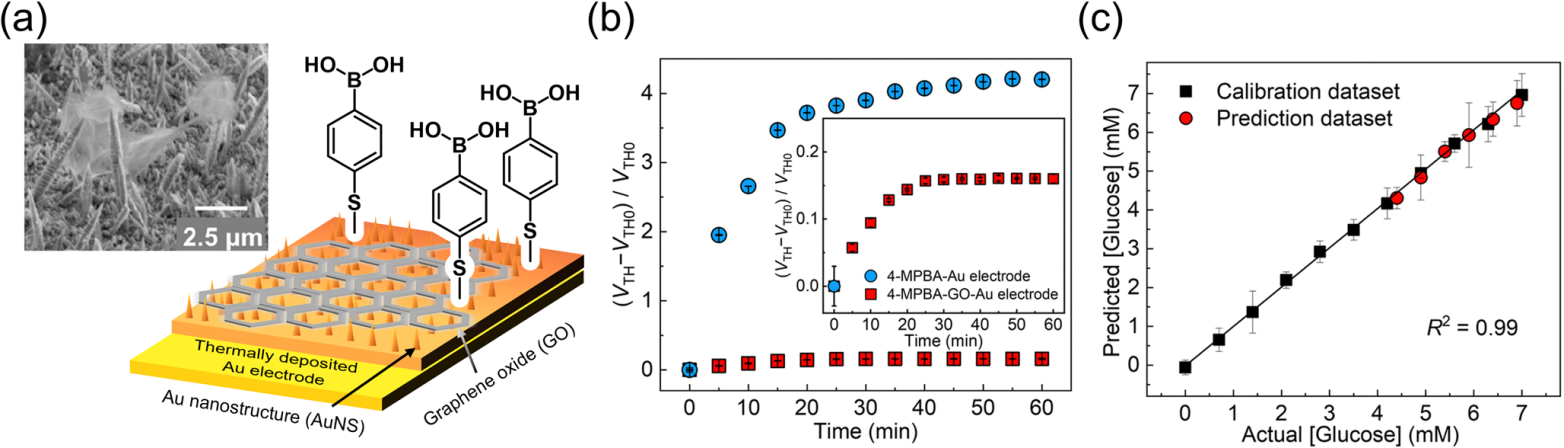
**a** Schematic illustration of an extended-gate electrode functionalized with Au nanostructures (AuNSs), graphene oxide (GO), and the phenylboronic acid (PBA) derivative for plasma glucose detection. The photograph represents the surface morphology of the extended-gate Au electrode characterized using the field-emission scanning electron microscopy, which indicates the AuNSs and thin-film GO layers. **b** Evaluation of the antifouling ability of the OFET in undiluted human blood plasma. The red squares and blue circles represent the PBA-attached extended-gate Au electrode with and without GO, respectively. The inset shows the enlarged figure for *V*_TH_ shifts. **c** Spike-and-recovery test results for glucose in human blood plasma. Reproduced with permission from the reference [18] Copyright 2023 The Royal Society of Chemistry

In real-sample analysis, interfering species such as immunoglobulin G and serum albumin present in human body fluids (e.g., blood plasma) can inhibit glucose detection due to physical adsorption of proteins onto the sensing electrode [51]. To date, blocking agents, such as bovine serum albumin and casein, have been used to avoid interfering effects caused by endogenous proteins in human body fluids [52]. In addition, surface modification approaches with long-chain polyethylene glycol monolayers have also been employed to suppress physical adsorption of proteins onto the sensing portion [53]. From the perspective of high-surface-area material, two-dimensional (2D) nanomaterials have attracted extensive research attention due to their exceptional structural and unique properties [54]. Among them, graphene oxide (GO) possesses multiple negative charges on its surface, which repel these negatively charged proteins via electrostatic interactions [55]. In this assay, gold nanostructures (AuNSs), GO, and the PBA derivative are modified on the extended-gate electrode surface (Fig. 4a) [18]. The AuNS layer with the needle-shaped architectures contributes to the enhancement of the accumulation of negatively charged GO layers [55]. Indeed, the surface morphology of the extended-gate electrode revealed the nanoarchitecture constructed by the AuNS and thin-film GO layers (Fig. 4a, inset). The chemical sensor, consisting of the OFET device and extended-gate electrode, showed almost no response to negatively charged proteins such as immunoglobulin G and serum albumin (Fig. 4b, red squares). Meanwhile, the transistor characteristics of the OFET device functionalized with the GO layer and PBA derivative were slightly shifted in the presence of undiluted human blood plasma, which was derived from the capture of plasma glucose by the PBA derivative as well as the suppression of the interfering protein adsorption by the GO layer (Fig. 4b, red squares, inset). In contrast, changes in the transistor characteristics in the presence of the above proteins were observed using the extended gate without modification of the GO layer (Fig. 4b, blue circles). These negligible changes indicated that the GO layer effectively suppressed nonspecific adsorption, demonstrating its antifouling ability. An OFET-based chemical sensor functionalized with the GO layer was used to detect plasma glucose. The obtained transistor characteristics corresponding to the glucose concentrations were used to establish a linear calibration line for the spike-and-recovery test (Fig. 4c, black squares). The recovery rates (red circles) were estimated to be 87–110%, and their accuracy was validated using a commercial dry-chemistry biochemical analyzer. Consequently, the integration of the 2D material with antifouling ability into the extended-gate OFET enabled the selective detection of plasma glucose owing to the suppression of interfering effects.

## Real-time monitoring using microfluidic technology

In chemical sensing, static and dynamic measurements are required depending on the sensing purpose. In particular, dysregulation of glucose levels can lead to serious diseases; therefore, facile real-time monitoring is crucial for diabetic patients [50]. From the perspective of molecular recognition materials, one of the inherent features of the PBA is reversibility based on dynamic covalent bonds, which enables the continuous detection of chemical information [19, 20]. In this regard, microfluidic technology has been extensively applied to analytical devices for continuous solution flow [56, 57], which enables full utilization of the reversible behavior of the PBA derivative for continuous glucose sensing. Owing to the dynamic behavior in sensing environments, time- and spatial-dependent concentration gradients of the analyte should be considered in sensor engineering. In this section, we introduce a methodology for real-time monitoring of glucose levels using microfluidic OFET-based glucose sensors.

**Fig. 5:**
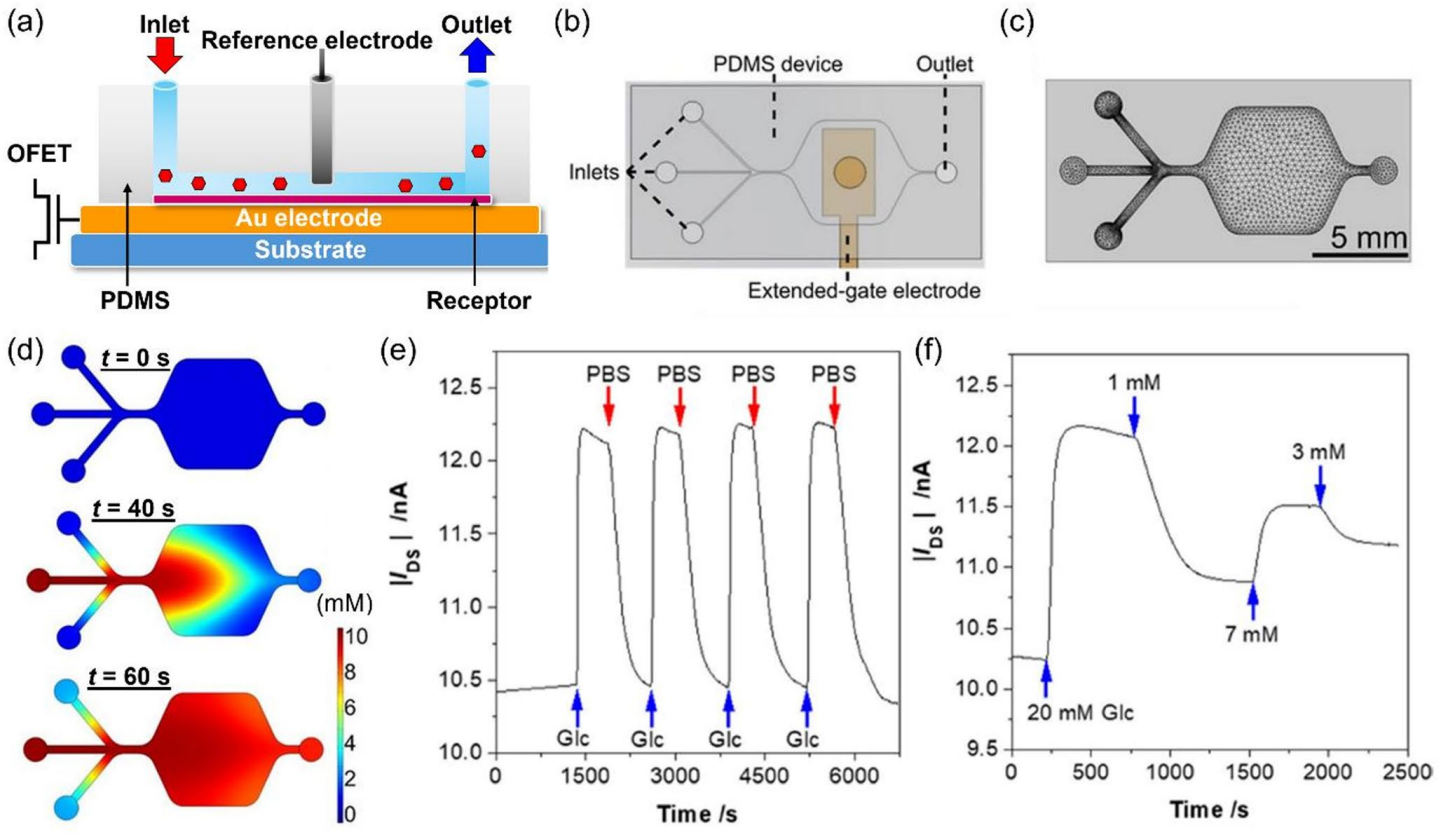
**a** Schematic illustration of the fabricated extended-gate organic field-effect transistor (OFET) sensor integrated with the microfluidic system. **b** Schematic top view of the microfluidic system combined with the extended-gate-type OFET, and **c** its mesh structure optimized by COMSOL simulations. **d** Simulation of time-dependent steady-state flow of phosphate buffer solution (PBS) in the microfluidic chamber. The flow rate was 25 μL min^−1^. **e** Time course of variation in *I*_DS_ with the injection of glucose solution (20 mM) and washing with PBS. **f** Simulated release and consumption of glucose with random variations in concentration. Reproduced with permission from the reference [19] Copyright 2020 Wiley–VCH GmbH

Herein, the microfluidic chamber was integrated into the extended-gate electrode functionalized with the PBA receptor (Fig. 5a) [19]. The microfluidic chamber is designed and optimized using simulation software, COMSOL Multiphysics^®^, according to the geometry of the chamber (including length, width, height, and crossing angle) and flow conditions [58, 59]. Indeed, the asymmetrical design of the chamber significantly contributed to effective flow uniformity [59–62]. The chamber contains three inlet lines for the alternative injection of target glucose and a washing solution (i.e., phosphate buffer solution [PBS]), and one outlet portion, which was designed to perform steady flow (Fig. 5b). The mesh structure of the microfluidic chamber was obtained via simulation with free triangular elements and a defined distribution size (Fig. 5c). In addition, the chamber geometry with biased corners in the mesh structure resulted in a highly uniform flow [61, 62]. As shown in Fig. 5d, the glucose diffused into the microfluidic chamber in a time-dependent manner within 60 s. Next, the OFET-based chemical sensor integrated into the microfluidic chamber was applied for reversible glucose detection. The sensor device exhibited an *I*_DS_ increase upon glucose injection, which was attributed to the formation of a negatively charged boronate ester on the extended-gate electrode surface [44, 45]. Alternating increases and decreases in *I*_DS_ were observed after the glucose injection and washing with PBS (Fig. 5e). This unique behavior was derived from a combination of the reversibility of the PBA receptor and the microfluidic technique. Moreover, the inherent reversibility of the OFET-based sensors enabled the monitoring of random increases and decreases in glucose concentration (Fig. 5f). This sensing behavior indicated the applicability of the microfluidic-based OFET device for monitoring glucose dynamics during cellular activity. In this context, the detectable concentration ranges of the sensor device against glucose matched glucose levels relevant for clinical diagnosis [50]. Taken together, the demonstration highlighted the potential of supramolecular receptors through the combination of an engineering approach based on microfluidics.

## Conclusion and perspective

Glucose is an essential biomarker to for examining health conditions; therefore, its accurate and facile monitoring is required. In the field of analytical chemistry, enzymatic approaches based on the lock-and-key principle have been widely used for selective glucose detection. However, traditional enzymatic sensors limit the design flexibility of enzyme libraries. In contrast, supramolecular receptors have emerged as robust synthetic recognition materials that can be designed based on molecular recognition chemistry. PBA derivatives enable selective glucose recognition through the strategic design of a recognition scaffold that engages the target glucose with multivalent interactions. In this mini-review, we have focused on PBA-based SAMs as a glucose-sensing interface, combined with an extended-gate-type OFET. An advantage of the extended-gate structure is the functionality of the sensing electrode. As a representative study, a two-dimensional material has been integrated into a sensing electrode functionalized with a PBA derivative to inhibit the physical adsorption of interfering proteins in blood plasma on the sensing electrode. Moreover, the reversible behavior of the PBA derivatives based on dynamic covalent bonds was highlighted by the real-time monitoring of glucose using a microfluidic chamber and an extended-gate OFET. To obtain an efficient fluidic performance, the microfluidic chamber and its flow dynamics were designed and optimized using simulations. The manufactured OFET-based microfluidic device exhibited a continuous response to glucose over a wide range of concentrations.

**Fig. 6:**
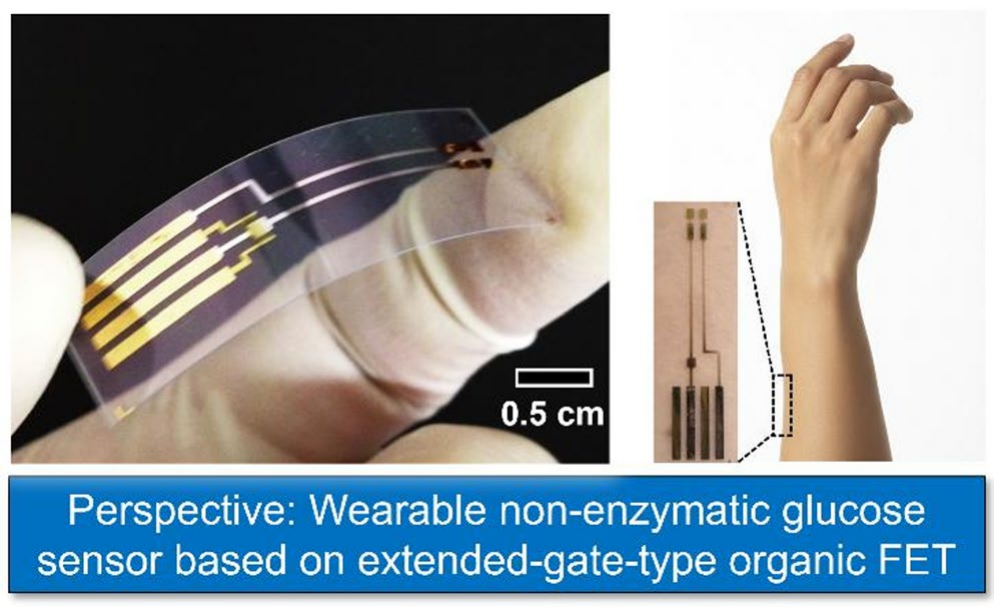
Perspective concept of a wearable non-enzymatic glucose sensor based on an extended-gate-type organic field-effect transistor (OFET). Reproduced with permission from the reference [31] Copyright 2019 Springer Nature

In this mini-review, we summarize a comprehensive methodology for multifunctionalized extended-gate electrodes tailored for specific sensing purposes. Even in the same sensor platform using an extended-gate OFET functionalized with the PBA receptor, additional sensor components and techniques, such as two-dimensional materials and microfluidic technology, have broadened the feasibility of non-enzymatic glucose sensors. One of the advantages of the extended-gate structure is the variety of sensor designs for practical applications [31]. In particular, the integrated design fabricated on a flexible substrate enables the development of wearable chemical sensors (Fig. 6) [5, 31]. We believe that the fusion of organic electronics, molecular recognition chemistry, and microfluidics will accelerate the realization of practical chemical sensor devices.

## Data Availability

This review article does not contain separate datasets.
